# Hemicrania from a Carious Tooth and Necrosed Root

**Published:** 1857-07

**Authors:** 


*Hemicrania from a Carious Tooth and Necrosed Root.*—We have received from our highly esteemed correspondent Dr. Levison, of London, the history of a very interesting case of hemicrania, caused by the irritation of a diseased tooth, but too late for insertion in the present number of the Journal. It will appear in our next issue; in the mean time we beg to call the attention of our readers to the first article in this number, from the pen of the same able writer, and who has already contributed so largely to enrich the literature of our profession. His long experience, and ability to trace the connection between cause and effects make all his articles on the pathology of the mouth peculiarly instructive and interesting, both to the student and practitioner of dental surgery.

